# Tyrosine 7.43 is important for mu-opioid receptor downstream signaling pathways activated by fentanyl

**DOI:** 10.3389/fphar.2022.919325

**Published:** 2022-09-02

**Authors:** Xiangyun Tian, Junjie Zhang, Shaowen Wang, Huan Gao, Yi Sun, Xiaoqian Liu, Wei Fu, Bo Tan, Ruibin Su

**Affiliations:** ^1^ State Key Laboratory of Toxicology and Medical Countermeasures, Beijing Key Laboratory of Neuropsychopharmacology, Beijing Institute of Pharmacology and Toxicology, Beijing, China; ^2^ Department of Medicinal Chemistry, School of Pharmacy, Fudan University, Shanghai, China; ^3^ Nanjing University of Chinese Medicine, Nanjing, China; ^4^ School of Pharmacy, Yantai University, Yantai, China

**Keywords:** *μ*-opioid receptor, molecular docking, cAMP, *β*-arrestin2 recruitment, signaling pathway, analgesia, molecular dynamics simulations

## Abstract

G protein–coupled receptors can signal through both G proteins and *ß*-arrestin2. For the *µ*-opioid receptor (MOR), early experimental evidence from a single study suggested that G protein signaling mediates analgesia and sedation, whereas *ß*-arrestin signaling mediates respiratory depression and constipation. Then, receptor mutations were used to clarify which residues interact with ligands to selectively regulate signals in a ligand-specific manner. However, there is no systematic study on how to determine these residues and clarify the molecular mechanism of their influence on signal pathways. We have therefore used molecular docking to predict the amino acid sites that affect the binding of ligands and MOR. Then, the corresponding sites were mutated to determine the effect of the structural determinant of MOR on G_i/o_ protein and *ß*-arrestin pathways. The pharmacological and animal behavioral experiments in combination with molecular dynamics simulations were used to elucidate the molecular mechanism of key residues governing the signaling. Without affecting ligand binding to MOR, MOR^Y7.43A^ attenuated the activation of both G_i/o_ protein and *ß*-arrestin signaling pathways stimulated by fentanyl, whereas it did not change these two pathways stimulated by morphine. Likewise, the activation peak time of extracellular regulated protein kinases was significantly prolonged at MOR^Y7.43A^ compared with that at MOR^wildtype^ stimulated by fentanyl, but there was no difference stimulated by morphine. In addition, MOR^Y7.43A^ significantly enhanced analgesia by fentanyl but not by morphine in the mice behavioral experiment. Furthermore, the molecular dynamics simulations showed that H6 moves toward the cellular membrane. H6 of the fentanyl–Y7.43A system moved outward more than that in the morphine–Y7.43A system. Y7.43 mutation disrupted hydrophobic interactions between W6.48 and Y7.43 in the fentanyl–Y7.43A system but not in the morphine–Y7.43A system. Our results have disclosed novel mechanisms of Y7.43 mutation affecting MOR signaling pathways. Y7.43 mutation reduced the activation of the G_i/o_ protein pathway and blocked the *ß*-arrestin2 recruitment, increased the H6 outward movement of MOR, and disrupted hydrophobic interactions. This may be responsible for the enhanced fentanyl analgesia. These findings are conducive to designing new drugs from the perspective of ligand and receptor binding, and Y7.43 is also expected to be a key site to structure optimization of synthesized compounds.

## Introduction

Opioids, such as morphine and the synthetic opioid fentanyl, are the mainstay analgesics for the treatment of acute and chronic pain in the clinic (Frank and Pollack, 2017; Eidson and Murphy, 2019). Opioid receptors (ORs) are the main target of opioids, and they are divided into three classical subtypes: *µ*-opioid receptor (MOR), *δ*-opioid receptor (DOR), and *κ*-opioid receptor (KOR) (Stein, 2016). They are widely distributed in the central and peripheral nervous systems (Kieffer et al., 1992; Chen et al., 1993; Furst et al., 2020). MOR is predominantly coupled to the G_i/o_ protein family, which transduces signals by inhibiting the production of cyclic adenosine 5′-monophosphate (cAMP) by adenylyl cyclase (AC) through G*_α_*-subunits and activating G protein–coupled inwardly rectifying potassium channels through G_*βγ*_ subunits, among other effectors. MOR signaling is regulated by the phosphorylation of intracellular C-terminal serine and threonine residues, which stabilizes the recruitment of *ß*-arrestin2, which leads to receptor internalization and additional kinase-driven signaling events. Upon the activation, there are two main downstream signaling pathways of MOR, namely, the G protein–dependent signaling pathway and the *ß*-arrestin2–mediated signaling pathway (Schmid et al., 2017), which are not only mechanically independent but also pharmacologically separated (Galandrin and Bouvier, 2006). The discovery of ligands that preferentially stimulate G protein or *ß*-arrestin2 signaling has led to the concept of a “biased agonist.”

It was reported that the G protein–dependent signaling pathway mediates pharmacological effects, such as analgesia and sedation, whereas the *ß*-arrestin–dependent signaling pathway mediates respiratory depression (Kelly, 2013). At present, the original suggestion that *ß*-arrestin2 signaling plays a key role in opioid-induced respiratory depression has been questioned (Kliewer et al., 2020; Bachmutsky et al., 2021). Even so, there is still a growing interest in the development of functionally selective G protein–coupled receptor (GPCR) ligands facilitated by hopes of producing more effective drugs with reduced side effects (Wacker et al., 2017). In the development of new opioid analgesic drugs, a milestone was a study published in 2005 on *ß*-arrestin2 KO mice that reported enhanced morphine analgesia with greatly diminished respiratory depression and constipation (Raehal et al., 2005). The first representative MOR ligand with G protein bias (oliceridine; brand name Olinvyk, previously TRV130) was obtained through a series of methods involving biological screening, analysis of structure–activity relationships, and structural modification (Chen et al., 2013; Soergel et al., 2014). However, recent clinical studies have failed to demonstrate a significantly broader therapeutic window in humans, when compared with the administration of morphine (Hertz, 2018). All the same, it is approved by the Food and Drug Administration for the treatment of moderate to severe acute pain (Tan and Habib, 2021). Then, TRV734, a close analog of TRV130, is an orally bioavailable G protein–biased MOR agonist developed by Trevena and currently in phase I trials (James et al., 2020; De Neve et al., 2021). The data suggested that TRV734 was safe and well tolerated. These studies add valuable information to the increasingly discussed concept of biased agonism at MOR.

Recent studies of the mechanism of opioids (DeWire et al., 2013) indicate that the mechanism of functional selectivity of ORs involves a wide range of interactions. Hothersall et al. (2017) found that the MOR^W7.35A^ downregulates *ß*-arrestin biased signaling of the ligand DAMGO, whereas the MOR^Y7.43F^ exhibits abrogated *ß*-arrestin signaling. Using residue mutation experiments, Sun et al. (2017) confirmed that DOR^W6.48^ significantly affects the *ß*-arrestin pathway but not G protein signaling. In the structure-based optimized synthesis of PZM21, Manglik et al. (2016) found that the disruption of hydrogen bond interactions between the urea and D3.32, Y7.43, and Q2.60 would result in a loss between 30- and 230-fold potency of the compound. For other GPCRs, residues S3.36 and Y7.43 of 5-HT_2C_ impacted ligand-binding pocket structure via hydrogen bond formation (Canal et al., 2011). These results suggest that the mechanism of functional selectivity and signal transduction of ORs is highly complex.

It remains elusive how the agonist binding influences receptor conformational dynamics and then affects the downstream signal pathway of the receptor. To detect the structural determinant controlling the effector coupling in MOR, receptor mutagenesis experiments were conducted here. It was demonstrated to be an effective approach to identifying the molecular switch that controls the signal transduction in a ligand-specific manner (Tschammer et al., 2011; Bock et al., 2012; Fowler et al., 2012; Fenalti et al., 2014; Wootten et al., 2016). It is important to note that evidence from a *β*_2_-adrenergic receptor mutation has indicated the residues in the ligand-binding pocket that can mediate agonist-selective effector coupling, thus supporting the hypothesis that qualitative differences in receptor signaling can be regulated at the level of ligand/receptor interactions (Woo et al., 2014). Likewise, mutations in MOR have been increasingly studied in recent years (Hothersall et al., 2017). However, few studies were conducted on how to determine the key residues in MOR that affect the downstream signaling pathways of the receptor. In this study, we first predict the key ligand-binding sites of MOR via molecular docking simulation, investigate the impact of site mutations on signaling pathways, and finally clarify the signal transduction mechanism via molecular dynamics (MD) simulation.

## Materials and methods

### Plasmids, antibodies, and chemicals

To construct MOR and Y7.43A mutant plasmids, full-length hOPRM1 cDNA was subcloned into the Tag-lite^®^ pT8-SNAP vector (Cisbio, Codolet, France). The hOPRM1 cDNA sequence was obtained from the NCBI website. The Vazyme^®^ Fast Mutagenesis Kit V2 (Vazyme Biotech Co., Ltd., Nanjing, China) was used to introduce the Y7.43A site mutation to the MOR recombinant plasmid using the following primers: F 5′-CTC​TAG​GTg​ctA​CAA​ACA​GCT​GCC​TCA​ACC​CA-3′ and R 5′-GTT​TGT​agc​ACC​TAG​AGC​AAT​GCA​GAA​GTG​CC-3′. The nucleotide sequences of mutant MOR were confirmed through DNA sequencing and sequence alignment.

Antibodies recognizing phosphorylated p44/42 MAPK (p-ERK1/2), ERK1/2, and *ß*-arrestin2 were purchased from Cell Signaling Technology (Frankfurt, HE, Germany). Polyclonal rabbit MOR antibody was purchased from Sigma-Aldrich (Darmstadt, Germany). GAPDH monoclonal antibody was purchased from Abcam (Cambridge, United Kingdom). HRP-coupled secondary antimouse and antirabbit antibodies were obtained from Jackson ImmunoResearch (Philadelphia, PA, United States).

Morphine, 7,8-Didehydro-4,5-epoxy-17-methyl-(5α,6α)-morphinan-3,6-diol hydrochloride, item no. 20020201 was purchased from Qinghai Pharmaceutical Factory (Xining, Qinghai, China). Fentanyl, N-Phenyl-N-[1-(2-phenylethyl) piperidin-4-yl] propanamide 2-hydroxypropane-1,2,3-tricarboxylate (1:1) was synthesized by the Military Medical Research Institute (Beijing, China). Forskolin was purchased from Sigma-Aldrich (Darmstadt, Germany). Naltrindole hydrochloride was purchased from Tocris Bioscience (Bristol, United Kingdom).

### Molecular modeling and docking

The *Homo sapiens* active MOR was built by homology modeling with the X-ray structure of *Mus musculus* active MOR (PDB code: 5C1M), using Discovery Studio 3.5. Align Sequences module was utilized to align *Homo sapiens* MOR and *murine* MOR sequences. All water molecules in the X-ray structure were retained. The Ramachandran plot was used to evaluate the validity of the homology models.

The fentanyl and morphine were docked into the 3D structure of *Homo sapiens* active MOR. Also, DAMGO was involved in docking as a reference agonist. The Induce Fit Docking module of the Schrödinger 3.5 software was used to dock them into the binding site of the active MOR. The two systems were subjected to Monte Carlo Multiple Minimum conformational searches using the OPLS_2005 force field, and the optimal conformations were chosen as binding conformations.

### Molecular dynamics simulations

The MD simulations were conducted using Gromacs 5.1.2 package. We built six systems for MD simulations: 1) MOR without ligand (Apo); 2) fentanyl-bound active MOR; 3) morphine-bound active MOR; 4) Y7.43A mutant MOR without ligand (Apo-Y7.43A); 5) fentanyl-bound active Y7.43A mutant MOR; and 6) morphine-bound active Y7.43A mutant MOR. All systems were embedded into the hydrated, equilibrated palmitoyloleoylphosphatidylcholine bilayer using the CHARMM-GUI interface. Sodium and chloride ions were added to neutralize systems and adjusted NaCl concentration to approximately 0.15 mol/l. All systems were minimized and gradually equilibrated in an NPT ensemble at 310 K and 1 bar. The MD simulations were conducted for 100 ns. All analyses of MD trajectories were conducted using tools implemented in the GROMACS 5.1.2 package.

### Homogeneous time-resolved fluorescence competitive binding assay

Homogeneous time-resolved fluorescence (HTRF) competitive binding assay was conducted according to the Tag-lite^®^ binding assay recommended protocol (Cisbio, Codolet, France). HEK293T cells were cultured in a DMEM medium (GIBCO, Grand Island, United States) containing 10% fetal bovine serum at 37°C with 5% CO_2_. They were grown on 60 mm dishes and transiently transfected with 4 μg WT/Y7.43A mutant MOR plasmid using Lipofectamine^®^ 3,000 (Invitrogen, Carlsbad, CA, United States) according to the manufacturer’s instructions. All cells were labeled in batch with 700 μl/dish SNAP-Lumi4-Tb and suspended in Tag-lite^®^ labeling buffer (TLB), and then, cells were dispensed in a 384 well small volume white microplate (Thermo Scientific Nunc, Waltham, MA, United States) at a density of 5,000 cells/well.

To determine the saturation binding constant K_d_ of the fluorescent ligand, which is a naltrexone derivative labeled with a red-emitting HTRF fluorescent probe (Cisbio, Codolet, France), cells were incubated with a series of concentrations (final concentration 0.1–100 nM) of the fluorescent ligand in TLB. Nonspecific binding signal wells were incubated with 100 nM Naltrindole hydrochloride. For competition binding experiments, cells were added fentanyl or morphine (final concentration 10^−14^ M–10^−4^ M) in competition against the 8 nM fluorescent ligand. All samples were mixed with a final volume of 20 μl and incubated at room temperature for 3 h. After incubation, HTRF signals were measured using Envision Multilabel Reader (PerkinElmer, Waltham, MA, United States) after excitation at 337 nm at both 620 and 665 nm emission, and the HTRF signal was calculated as a two-wavelength signal ratio: [intensity (665 nm)/intensity (620 nm)].

### Time-resolved cyclic adenosine 5′-monophosphate measurement assay

Measurement of ligand-induced cAMP production in HEK293T cells transiently expressing MOR^wildtype^/MOR^Y7.43A^ was performed using the GloSensor cAMP biosensor (Promega, Madison, WI, United States) according to the manufacturer’s protocol. In a word, HEK293T cells were grown on 60 mm dishes and transiently cotransfected with 2 μg MOR^wildtype^/1.58 μg MOR^Y7.43A^ plasmid and 1.4 μg pGloSensor-22 F cAMP plasmid (Promega, E2301). After 8 h of transient transfection, the cells were digested by trypsin and distributed evenly into the white 96-well poly-D-lysine-coated plate (Thermo Fisher Scientific Nunc, Roskilde, Denmark). After 24 h, cell media were replaced by 90 ml of fresh full DMEM with 2% v/v GloSensor cAMP Reagent (Promega, E1290) and incubated for 120 min at 37°C. Firefly luciferase activity was measured using Envision Multilabel Reader. After initial measurement of the baseline signal for 15 min, cells were stimulated with 10 μl of 10× drug solution of the MOR agonist fentanyl or morphine (final concentration 10^−12^ M–10^−4^ M) for 10 min. Then, 1 μl of forskolin (final concentration 10 μM) solution was added for 15 min, and the real-time chemiluminescent signal was measured at once. The inhibition of cAMP production was calculated using the baseline signal, drug response signal, and forskolin signal. Then, sigmoidal dose–response curves with variable slope (three-parameter logistic regression) were plotted. Results are from three independent experiments.

### Time-resolved *ß*-arrestin2 recruitment assay

Coupling between MOR and *ß*-arrestin2 was monitored using a NanoBiT protein:protein interaction assay (Promega, M2014). MOR^wildtype^ and MOR^Y7.43A^ were cloned into pBiT2.1-N [TK/SmBiT] vector and *ß*-arrestin2 into pBiT1.1-C [TK/LgBiT] vector. For the negative control, the MOR-SmBiT construct was substituted with the NanoBiT negative control vector (HaloTag-SmBiT). The positive control consisted of SmBiT-PRKACA and LgBiT-PRKAR2A vectors; 2.4 μg WT/Y7.43A-SmBiT together with 0.8 μg LgBiT-β-arrestin2 were used to transiently transfect HEK293T cells in 60 mm dish using Lipofectamine 3,000 according to the manufacturer’s protocol. After 8 h, the cells were digested by trypsin and distributed evenly into the white 96-well poly-D-lysine-coated plate. The next day, 30 min prior to the assay, the culture medium was substituted for 100 μl/well serum-free Opti-MEM medium (GIBCO, Grand Island, United States). The nanoluciferase activity was measured using Envision Multilabel Reader. After the initial measurement of the background signal, 25 μl/well Nano-Glo^®^ Live Cell Assay System (Promega, N2013) was added and cells were equilibrated and basal luciferase activity was measured for 15 min. Then, cells were stimulated with 10 μl of 13.5× drug solution of the MOR agonist fentanyl or morphine (final concentration 10^−12^ M–10^−5^ M), and a chemiluminescent signal was measured for 15 min (5 min intervals). The *ß*-arrestin2 recruitment was calculated using the baseline signal and the drug response signal. Then, sigmoidal dose–response curves with variable slope (three-parameter logistic regression) were plotted. Results are from three independent experiments.

### Quantitative real-time polymerase chain reaction analysis

The transfected samples for the *ß*-arrestin2 recruitment assay were seeded in six-well plates. The next day, RNA was extracted using TRIzol (Sigma-Aldrich, St. Louis, MO, United States). cDNA was synthesized using the RevertAid first strand cDNA synthesis kit (Thermo Fisher Scientific Baltics UAB, Vilnius, Lithuania). Relative MOR^wildtype^ and MOR^Y7.43A^ expressions were determined on 250 ng cDNA using SYBR Green quantitative polymerase chain reaction (PCR) reagents (Thermo Fisher Scientific Baltics UAB, Vilnius, Lithuania), normalized to GAPDH expression levels. The forward primer of MOR^wildtype^/MOR^Y7.43A^ was GCC​CTT​CCA​GAG​TGT​GAA​TTA​C, the reverse primer of MOR^wildtype^/MOR^Y7.43A^ was GTG​CAG​AGG​GTG​AAT​ATG​CTG, the forward primer of GAPDH was AGG​TCA​TCC​CAG​AGC​TGA​ACG, and the reverse primer of GAPDH was TCA​GAT​GCC​TGC​TTC​ACC​AC.

### Western blot analysis

After 24 h of transient transfection of MOR^wildtype^ and MOR^Y7.43A^ plasmids into HEK 293T cells, the transfected cells were starved in serum-free DMEM medium for 2 h. Then, the cells were exposed to 10 μM fentanyl and morphine for an indicated time. Immediately following stimulation, all cells were lysed in an ice-cold RIPA lysis buffer (50 mM pH 7.4 Tris, 150 mM NaCl, 1% Triton X-100, 1% sodium deoxycholate, 0.1% SDS) supplemented with protease inhibitor cocktail and phosphatase inhibitor (Roche, Basel, Switzerland). Protein samples were resolved by 10% SDS-PAGE and transferred onto PVDF membranes (Millipore, Carrigtwohill, Co. Cork, Ireland). After the membranes were blocked with 5% milk, they were incubated overnight with primary antibodies against p-ERK1/2, ERK1/2, MOR, and GAPDH at 4°C. The membranes were washed with TBST and incubated with the secondary antibody for 1 h at room temperature. The immune complexes were detected using western blot detection reagents (Millipore, Burlington, MA, United States).

### Mice

MOR knockout mouse strain B6.129S2-*Oprm1* ^*tm1Kff*^/J (MOR^−/−^, RRID: IMSR_JAX:007,559) was obtained from The Jackson Laboratory. Mice were genotyped via PCR analysis of genomic tail-biopsy DNA using the following primers: 5′-GCC​AGA​GGC​CAC​TTG​TGT​AG-3′, 5′-ATC​TTC​ACC​CTC​TGC​ACC​AT-3′, and 5′-TGC​TGG​GCT​CAA​GCT​TTA​AT-3’. JAX™C57BL/6J (RRID: IMSR_JAX:000,664) mice from The Jackson Laboratory were used as wild-type control. Animals were housed four to six per cage under a 12 h light–dark cycle (lights on at 8 a.m.) and ventilated at a controlled temperature with *ad libitum* access to food and water. In hot plate experiments, we used female mice aged 8–16 weeks because the genitals of male mice touched the hot plate, which can produce an allergic reaction. Each animal was used only once for each dose and each drug tested. Studies were conducted in parallel such that age-matched mice received the same drug treatment at the same time. All experimental protocols were approved by and conducted in accordance with the Animal Care and Use Committee of the Beijing Institute of Pharmacology & Toxicology (Beijing, China).

### AAV package, intracerebroventricular injection, and expression in central nervous system

The AAV (BrainVTA Co., Ltd., Wuhan, Hubei, China) that overexpressed MOR^wildtype^/MOR^Y7.43A^ was rAAV2-EF1α-EGFP-WPRE-hGH polyA, AAV-PHP.eB vector containing MOR^wildtype^/MOR^Y7.43A^ insert of mice.

The heads of mice were firmly fixed by the thumb and index finger. A 25 µl tip microsyringe was used to slowly inject AAVs (5 µl) into the right cerebral lateral ventricle (at coordinates −1.0 mm mediolateral, −0.5 mm anteroposterior from Bregma; −2.0 mm dorsal–ventral from the skull) (Craft et al., 2005).

Twenty-one days following virus injection, mice were sacrificed through transcardial perfusion of deeply anesthetized mice using intraperitoneal-injected 1% sodium pentobarbital. Mice were perfused with 0.9% saline followed by 4% paraformaldehyde in 0.1 M PO_4_, after which whole-brain dissections were performed. Brains were postfixed overnight in 4% paraformaldehyde in 0.1 M PO_4_ followed by incubation for a minimum of 48 h in 30% sucrose in 0.1 M PO4 (Kofoed et al., 2021). Fifty-micrometer sections were cut on a sliding microtome with a freezing stage (Leica Biosystems, Buffalo Grove, IL, United States) (Rincon et al., 2018). Sections were collected in a 24-well plate filled with antifreeze (PBS: glycerol: ethylene glycol = 5:2:3) and stored in a refrigerator at −20°C and were scanned using Olympus VS.120.

### Hot plate test

Opiate effects on paw withdrawal latencies were assessed as the time to response (licking hind paw, quick pedaling, and jumping (s)) after placement on a hot plate maintained at 55 ± 0.5°C (Shao et al., 2020). To avoid tissue damage, we used a 60 s cutoff. The hot plate test was conducted 15 min (fentanyl) or 30 min (morphine) after drug administration and expressed as the percent maximum possible effect (%MPE), calculated as follows: %MPE = [(drug response latency−basal response latency)/(60 s−basal response latency)] ×100. For the analgesic model, after testing basal response time, mice were subcutaneously (s.c.) injected with fentanyl (0.1 mg/kg) or morphine (10 mg/kg). The time courses for antinociception were determined at various time points after drug treatment.

### Statistical analysis

Data are presented as mean ± SEM of at least three individual experiments. For HTRF competitive binding assay, concentration–response curves were fit to one-site binding models provided in GraphPad Prism software to determine K_d_ and K_i_. For EC_50_ values of concentration–response curves, the best-fit line was generated following nonlinear regression analysis, as described in each section above. The results of the HTRF competitive binding assay, cAMP measurement assay, and *ß*-arrestin2 recruitment assay were evaluated via an unpaired two-tailed *t*-test. For the hot plate test experiment, data were analyzed using two-way ANOVA, followed by Bonferroni’s post hoc tests. In all the experiments, a value of *p* < 0.05 was considered significant and all calculations were performed using GraphPad Prism 8.0 software.

## Results

### Y7.43 site was involved in the interaction of fentanyl and morphine with *µ*-opioid receptor

**FIGURE 1:**
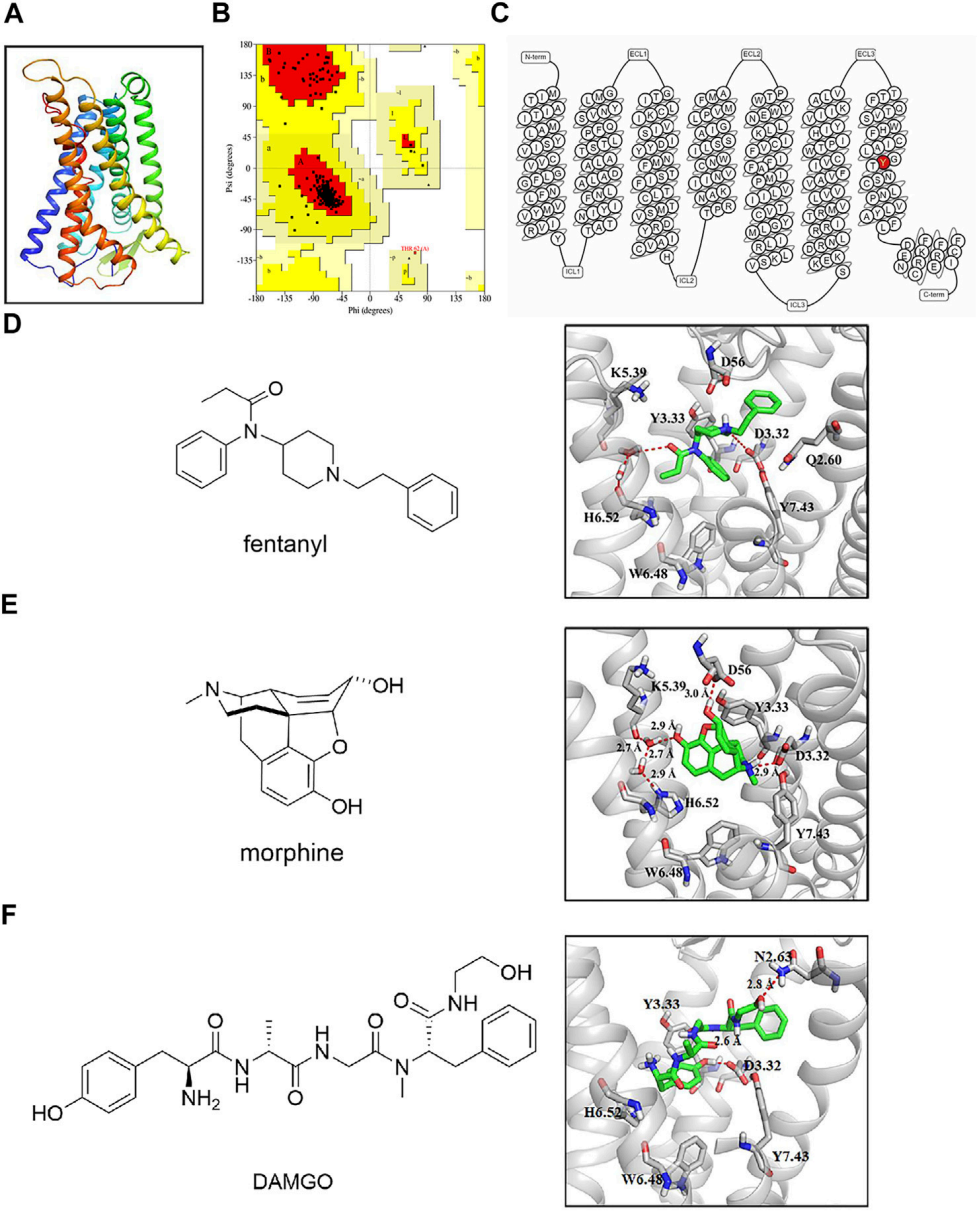
Binding mode of agonists with active µ-opioid receptor (MOR). **(A)** three-dimensional structure model of *Homo sapiens* MOR. **(B)** Ramachandran plot of active MOR model from active *Mus musculus* MOR. **(C)** snake diagram of MOR with residue Y7.43 highlighted in magenta. Image created from www.gpcrdb.org. **(D–F)** binding mode of agonists with active MOR. Active MOR is shown in gray ribbons; residues are shown as a gray ball–stick model. Hydrogen bond is shown as a dashed line. Agonists are shown as a green ball–stick model.

The 3D model of *Homo sapiens* active OR was obtained by homology modeling with the X-ray structure of *Mus musculus* active MOR (PDB code: 5C1M) and is shown in Figure 1A. The sequence identity and sequence similarity between active *Homo sapiens* MOR and *murine* MOR were 95.2% and 99.6%, respectively. In the Ramachandran plot, 96.7% amino acids were located in the most favored regions and 3.3% amino acids were located in additional allowed regions (Figure 1B). The snake diagram of MOR with residue Y7.43 highlighted is shown in Figure 1C.

The molecular docking diagram showed residues of MOR that interact with agonists. The most reasonable conformation of fentanyl in MOR was selected in our docking process based on the following criteria: 1) the lowest binding free energy and 2) the most frequently binding pose. The docking pose of fentanyl was subsequently optimized through MD simulation. It is consistent with the message-address concept: the protonated amine of the piperidine ring in fentanyl formed electrostatic interaction with the acidic D3.32 (message site), and the n-alkyl phenyl formed extensive hydrophobic contacts with transmembrane helices (TM) 2/3 (address site). The result was consistent with de Waal’s studies (de Waal et al., 2020). Also, the DAMGO participates in the docking process as a reference agonist. As shown in the binding mode with MOR in Figures 1D–F, the protonated nitrogen of three compounds engaged in a strong salt bridge with the carboxyl oxygen atom of D3.32, and the oxygen of amide of fentanyl and the phenolic hydroxyl of morphine participated in the hydrogen-bonding network with H6.52 via two water molecules. In addition, D3.32 forms the bidentate hydrogen bonds with Y7.43. As a critical site, D3.32 formed conserved ionic bonds with the protonated ligands. This interaction was observed in structures of the MOR, DOR, KOR, and nociceptin receptor bound to ligands of different scaffolds (Manglik et al., 2016). Therefore, Y7.43 was selected for a mutation to study the mechanism of activating signaling pathways.

### Y7.43 site mutation did not change the affinity of ligands in binding with *µ*-opioid receptor

The binding affinity of fentanyl and morphine in membranes from cells expressing MOR^wildtype^/MOR^Y7.43A^ was estimated using a competitive TR-FRET (time-resolved fluorescence resonance energy transfer)–based assay (Perez-Aguilar et al., 2013), and derived pK_i_ values are compiled in Table 1. From these experiments, the K_d_ of fluorescent ligand was estimated as (13.81 ± 2.69) and (35.49 ± 2.97) nM, respectively, at MOR^wildtype^ and MOR^Y7.43A^. In addition, we determined the expression of MOR^wildtype^ and MOR^Y7.43A^. There was no statistical difference in both whole cell and cell membrane via western blot and fluorescence labeling, respectively (Supplementary Figures S1A,B). In conclusion, the relative affinity of morphine at MOR^Y7.43A^ was 101% of MOR^wildtype^ pK_i_, and the pK_i_ value of fentanyl was 91% of MOR^wildtype^ pK_i_.

**TABLE 1:** Binding affinity of the agonists of wild type and Y7.43A mutant µ-opioid receptor

Agonist	Affinity (pK_i_ nM)	
	WT	Y7.43A
Fentanyl	5.87 ± 0.06	5.33 ± 0.06*
Morphine	5.59 ± 0.01	5.65 ± 0.11

Affinity is represented as average pKi ± standard error of the mean (*n* = 3) derived from fluorescent ligand competition binding in membranes from cells expressing WT and Y7.43A mutant MOR from three experiments performed in duplicate.

*p < 0.05, compared with WT group.

WT, wild type; MOR, µ-opioid receptor.

### Y7.43 site mutation reduced the activation of *µ*-opioid receptor–mediated G_i/o_ protein–dependent signaling stimulated by fentanyl

**FIGURE 2:**
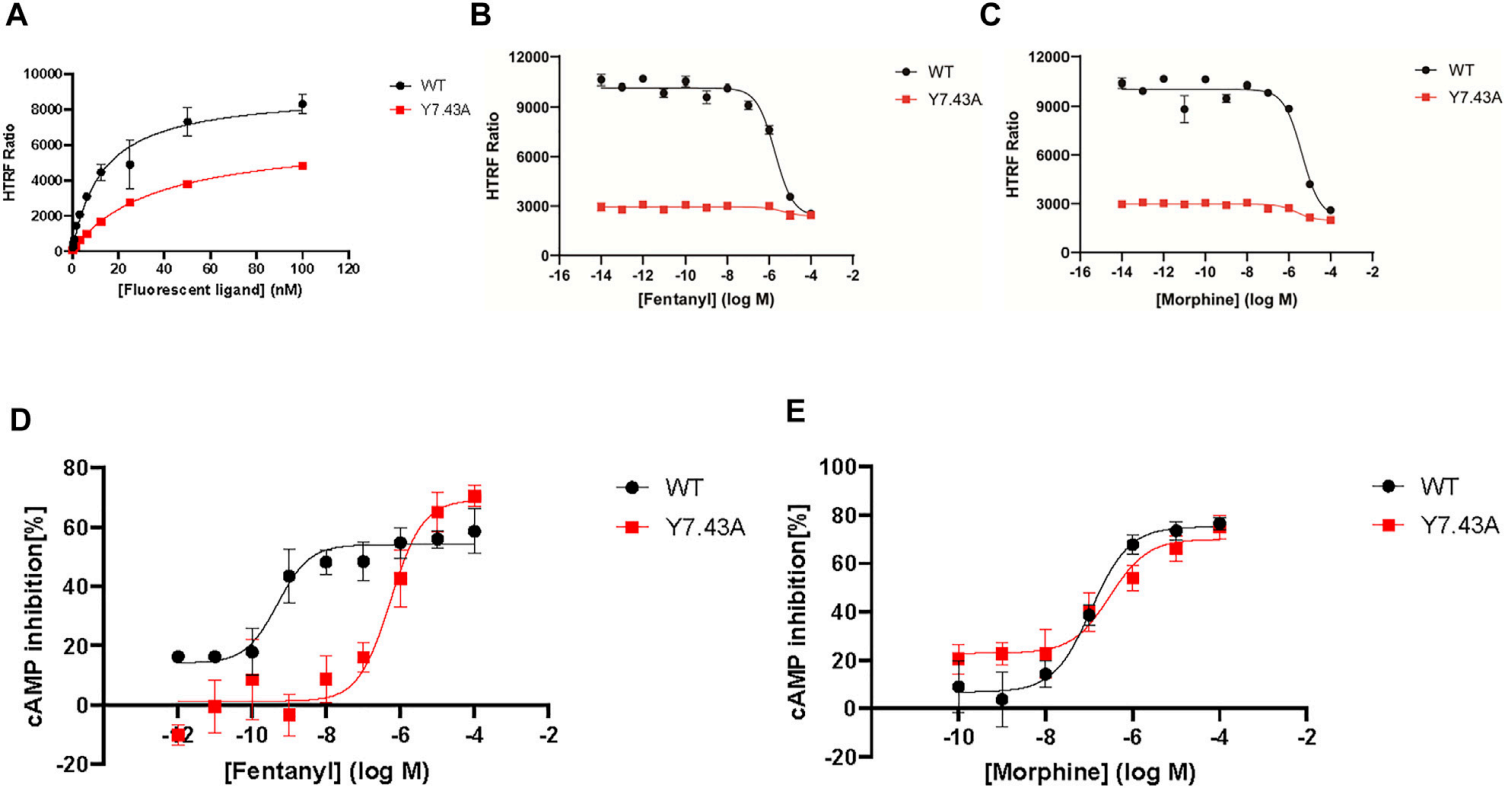
Effect of fentanyl and morphine on 10-μM forskolin-stimulated cyclic adenosine 5′-monophosphate (cAMP) accumulation in wild type (WT) and Y7.43A mutant µ-opioid receptor (MOR). **(A)** fluorescent ligand saturation binding of WT and Y.43A mutant MOR. **(B)** homogeneous time-resolved fluorescence (HTRF) competition binding of fluorescent ligand to fentanyl (WT: pK_i_ = 5.87 ± 0.06 nM; Y7.43A: pK_i_ = 5.33 ± 0.06 nM). **(C)** HTRF competition binding of fluorescent ligand to morphine (WT: pK_i_ = 5.59 ± 0.01 nM; Y7.43A: pK_i_ = 5.65 ± 0.11 nM). **(D,E)** dose–response curves for fentanyl- and morphine-induced cAMP production for WT (fentanyl: EC_50_ = 0.53 ± 0.15 nM, E_max_ = 54.18 ± 1.86%; morphine: EC_50_ = 119.94 ± 52.02 nM, E_max_ = 75.28 ± 3.09%) and Y7.43A mutant MOR transfected cells (fentanyl: EC_50_ = 546.67 ± 22.78 nM, E_max_ = 69.00 ± 0.58%; morphine: EC_50_ = 291.13 ± 181.44 nM, E_max_ = 69.86 ± 3.93% compared with WT). Data represented as mean ± standard error of the mean from three independent experiments; the statistical significance of differences between WT and Y7.43A mutant MOR analyzed using an unpaired two-tailed *t*-test.

To explore the effect of Y7.43 mutation on the activity of the G_i/o_ protein pathway downstream of MOR, we set up a GloSensor cAMP bioassay by transiently transfecting the plasmid pGloSensor-22F™ into HEK293T cells (Gilissen et al., 2015). The cAMP production was detected by this bioassay. The results showed that EC_50_ of fentanyl was (546.67 ± 22.78) nM for MOR^Y7.43A^, which is much higher than that observed for MOR^wildtype^ (0.53 ± 0.15) nM. By contrast, the EC_50_ of morphine was (291.13 ± 181.44) nM for MOR^Y7.43A^, with no significant difference between the MOR^Y7.43A^ and MOR^wildtype^ (119.94 ± 52.02) nM (Figures 2D, E, and Table 2). Next, we determined the expression of MOR^wildtype^ and MOR^Y7.43A^. There was no statistical difference in both whole cell and cell membrane based on western blot and fluorescence labeling, respectively (Supplementary Figures S1C,D), indicating that the MOR^Y7.43A^ could weaken the activity of the G_i/o_ protein pathway stimulated by fentanyl.

**TABLE 2:** Activation parameters of test agonists to stimulate µ-opioid receptor–mediated G_i/o_ protein and *ß*-arrestin signaling pathways

Agonist	WT		Y7.43A					
	cAMP		β-arrestin2		cAMP		*β*-arrestin2	
	EC_50_ (nM)	E_max_ (%)	EC_50_ (nM)	E_max_	EC_50_ (nM)	E_max_ (%)	EC_50_ (nM)	E_max_ (%)
Fentanyl	0.53 ± 0.15	54.18 ± 1.86	6.75 ± 2.01	16,521.33 ± 1446.39	546.67 ± 22.78****	69.00 ± 0.58***	—	—
Morphine	119.94 ± 52.02	75.28 ± 3.09	—	—	291.13 ± 181.44	69.86 ± 3.93	—	—

Half maximal effective concentration (EC_50_) and maximal effect (E_max_) derived from the concentration–effect analysis in the cAMP and *ß*-arrestin assay. average ± standard error of the mean, *n* = 3.

—: no value. *p < 0.05, **p < 0.01, ***p < 0.001, ****p < 0.0001 compared with WT group.

WT, wild type; cAMP, cyclic adenosine 5′-monophosphate.

### Y7.43 site mutation blocked *ß*-arrestin2 recruitment to *µ*-opioid receptor and affected downstream signaling

**FIGURE 3:**
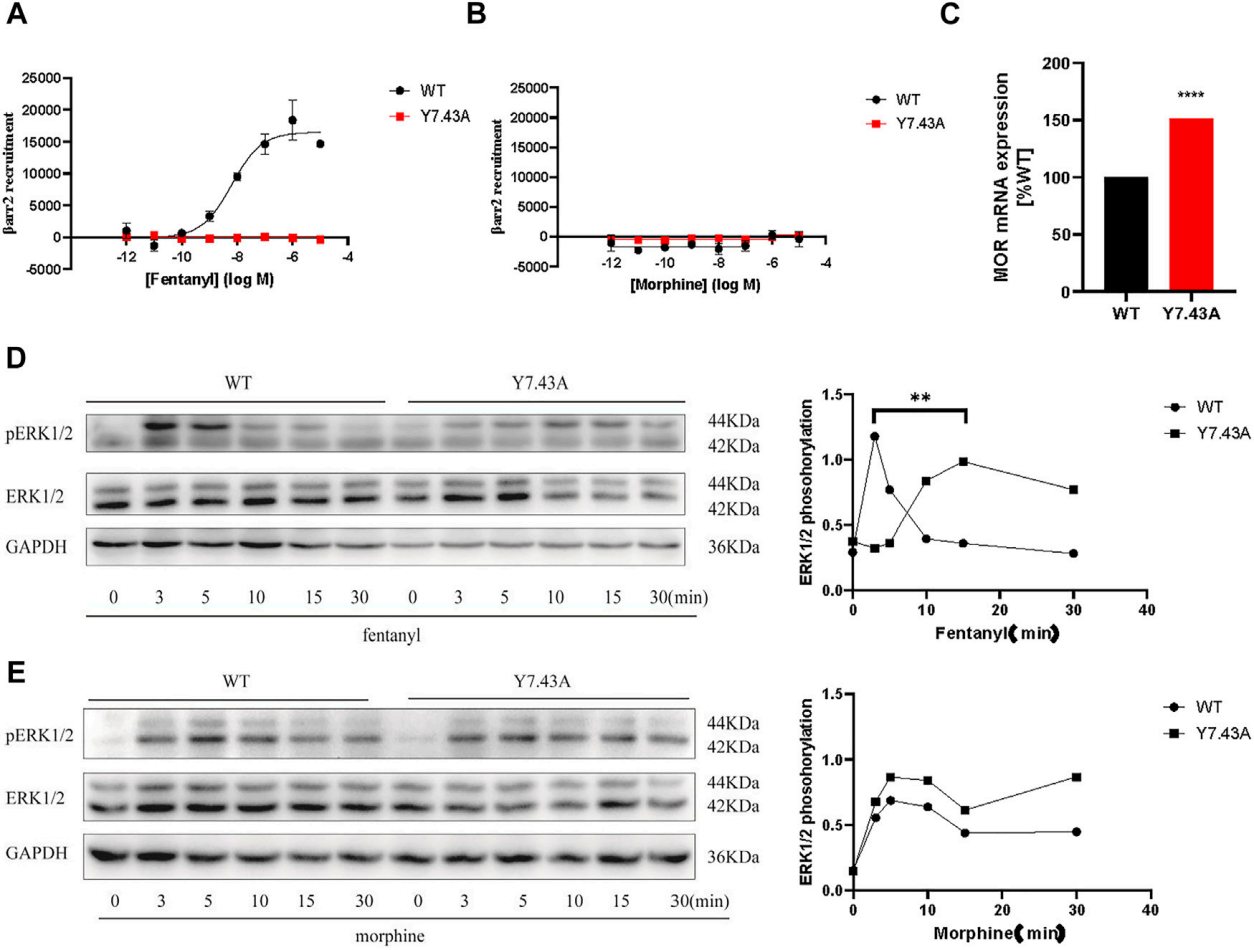
Effect of *ß*-arrestin2 recruitment stimulated by fentanyl and morphine in wild type (WT) and Y7.43A mutant µ-opioid receptor (MOR). **(A,B)** dose–response curves for fentanyl- and morphine-induced *ß*-arrestin2 recruitment for WT (fentanyl: EC_50_ = 6.75 ± 2.01 nM, E_max_ = 16,521.33 ± 1446.39) and Y7.43A mutant MOR transfected cells. **(C)** receptor cell surface expression via quantitative real-time polymerase chain reaction assay. Data represented as mean ± standard error of the mean from three independent experiments; the statistical significance of differences between WT and Y7.43A mutant MOR analyzed using an unpaired two-tailed *t*-test (p**** <0.0001). **(D,E)** representative western blots and quantification of ERK1/2 phosphorylation by WT and Y7.43A mutant MOR before (−) and after (+) stimulation by **(D)** fentanyl and **(E)** morphine in a time-course experiment; GAPDH used as a loading control; the quantification of ERK1/2 phosphorylation refers to the pERK/ERK ratio. * indicates statistically significant differences at the peak time point of ERK1/2 activation compared with WT.

To further explore Y7.43 site mutation effects on the *ß*-arrestin–dependent signaling pathway, we focused on the interaction between MOR and *ß*-arrestin2 using NanoBiT assay. The results showed that MOR^Y7.43A^ markedly inhibited the *ß*-arrestin2 recruitment induced by fentanyl (Figure 3A), whereas MOR^Y7.43A^ did not change the status of *ß*-arrestin2 induced by morphine. That is, MOR^wildtype^ and MOR^Y7.43A^ failed to recruit *ß*-arrestin2 (Figure 3B). We also confirmed that the MOR^wildtype^ and MOR^Y7.43A^ were successfully expressed using quantitative real-time PCR analysis (Figure 3C). ERK plays a crucial role in the regulation of MOR signaling and opioid analgesia (Ruan et al., 2019; Zhang et al., 2020). MOR-mediated ERK1/2 activation is involved not only in G protein–dependent signaling but also in *ß*-arrestin–dependent signaling (Rozenfeld and Devi, 2007). Therefore, we monitored the magnitude and duration of ERK1/2 phosphorylation stimulated by fentanyl and morphine. For MOR^wildtype^ and MOR^Y7.43A^ induced by morphine, the activation peaks of ERK1/2 were similar (Figures 3D,E). Combined with the similar degree of G_i/o_ protein signaling pathway activation, it was suggested that morphine-mediated ERK1/2 phosphorylation was G protein–dependent rather than relying on *ß*-arrestin. The results also showed that the peak activation of MOR^wildtype^ ERK1/2 by fentanyl was at 3 min time point, whereas MOR^Y7.43A^ prolonged the activation peak time that appeared at 15 min. Combined with the fact that MOR^Y7.43A^ significantly reduced the activity of the G_i/o_ protein pathway and inhibited the *ß*-arrestin2 recruitment induced by fentanyl, this indicates that there is a relationship between ERK1/2 activation and *ß*-arrestin2 downstream of MOR stimulated by fentanyl. These results demonstrated that Y7.43 may be an important amino acid site affecting fentanyl-mediated *ß*-arrestin2 recruitment and ERK1/2 activation downstream of MOR.

### *µ*-opioid receptor^Y7.43A^ enhanced fentanyl analgesia

**FIGURE 4:**
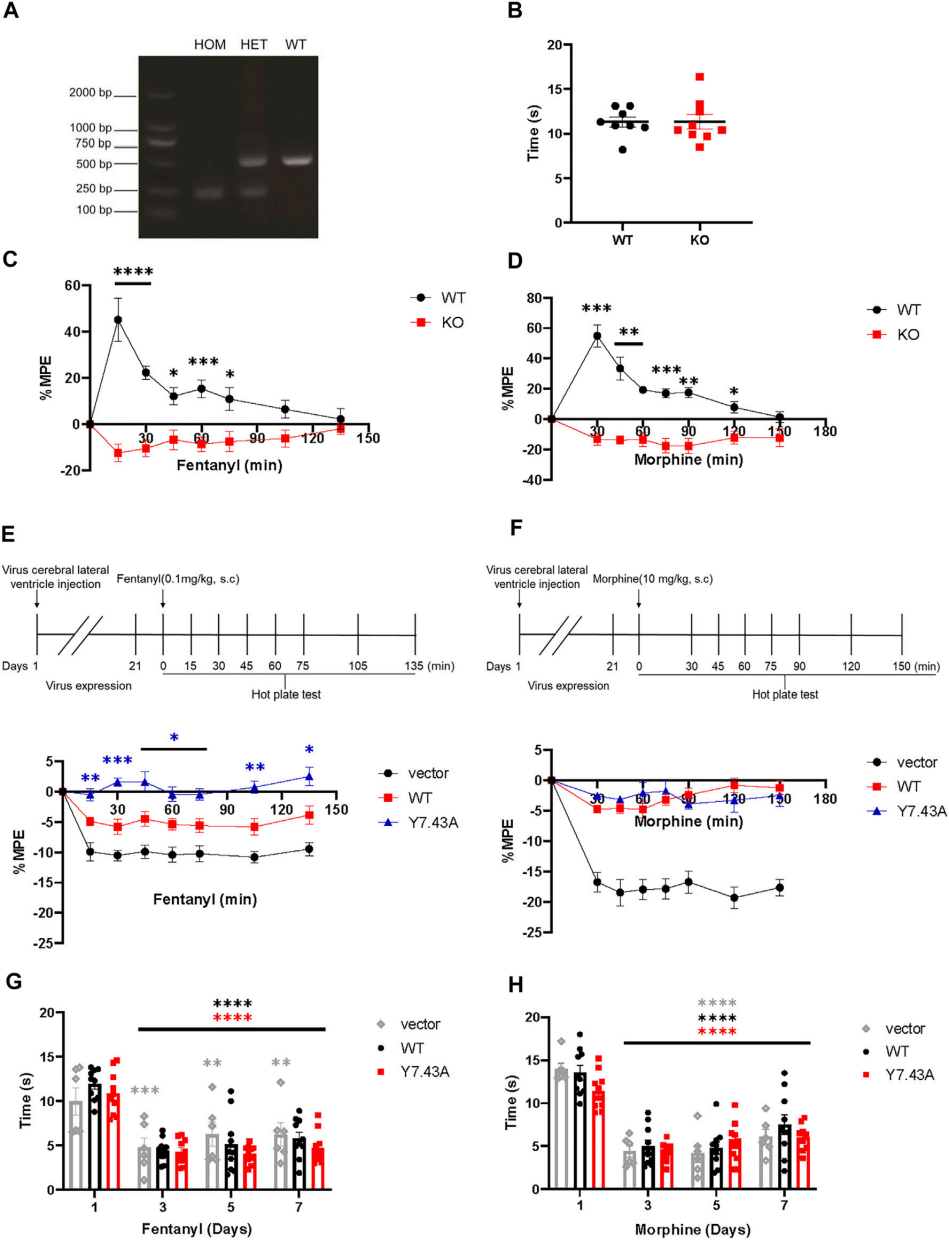
Results of hot plate test. **(A,B)** verification of µ-opioid receptor (MOR) KO mice. **(A)** mice were genotyped via polymerase chain reaction analysis of genomic tail-biopsy DNA using the following primers: 5′-GCC​AGA​GGC​CAC​TTG​TGT​AG-3′, 5′-ATC​TTC​ACC​CTC​TGC​ACC​AT-3′, and 5′-TGC​TGG​GCT​CAA​GCT​TTA​AT-3′. **(B)** baseline of pain threshold of MOR KO or JAX™C57BL/6J wild type (WT) control mice (WT, 11.30 ± 1.48 s, *n* = 8; KO, 11.33 ± 2.39 s, *n* = 9). **(C,D)** antinociceptive effect of MOR KO mice given 0.1 mg/kg fentanyl **(C)** and 10 mg/kg morphine **(D)** was completely opposite to WT mice. Significant differences of analgesia between MOR KO and WT groups are indicated by asterisks: *p < 0.05, **p < 0.01, ***p < 0.001, ****p < 0.0001 (*n* = 7). Two-way analysis of variance (ANOVA) with Bonferroni post hoc test; MOR KO mice aged 8–16 weeks were injected with AAV-wildtype and AAV-Y7.43A allowed 21 days for virus expression. **(E,F)** acute antinociceptive response measured in the mice hot plate test 15 min (fentanyl) or 30 min (morphine) after drug administration. Nociceptive latencies were defined by licking, stomping, or jumping and are reported as percent maximum possible effect with a 60 s cutoff. Analgesic time course of acutely administered fentanyl **(E)**, (*F*_genotype_ (2, 27) = 59.79, *p* < 0.0001; *F*_time_ (5.310, 131.2) = 11.22, *p* < 0.0001) or morphine **(F)**, (*F*_genotype_ (2, 27) = 71.41, *p* < 0.0001; *F*_time_ (5.151, 131.7) = 20.58, *p* < 0.0001) was repeated after fentanyl and morphine administration, respectively, for the indicated times. Significant differences of analgesia between WT and Y7.43A groups are indicated by asterisks: *p < 0.05, **p < 0.01, ***p < 0.001 (*n* = 10). Two-way ANOVA with Bonferroni post hoc test. **(G,H)** administration drugs for 7 consecutive days, the change of basal pain threshold in MOR KO mice injected with AAV-vector, AAV-WT, and AAV-Y7.43A. Mice were given fentanyl and morphine twice daily (8 a.m. and 6 p.m.) for 7 days, and then, baseline pain thresholds were measured before drug administration on the first, third, fifth, and seventh days. Basal pain thresholds on the third, fifth, and seventh days were significantly lower than on the first day (*p < 0.05, **p < 0.01, ***p < 0.001, ****p < 0.0001, *n* = 10). Two-way ANOVA with Bonferroni post hoc test.

To study the Y7.43 site mutation for analgesia *in vivo*, we conducted the hot plate test in mice expressing MOR^Y7.43A^. We created suitable mutant mouse models, which were injected with MOR^wildtype^/MOR^Y7.43A^ AAV in the right cerebral lateral ventricle of MOR KO mice. The absence of MOR protein expression was confirmed by the mice-tail genotyping (Figure 4A). We also confirmed that MOR KO mice injected with fentanyl and morphine had the completely opposite analgesic effect to WT mice given the same dose, whereas WT and KO mice showed no difference in basic response to hot plate (WT, 11.30 ± 1.48 s, *n* = 8; KO, 11.33 ± 2.39, *n* = 9; Figures 4B–D).

After 21 days of AAV injection, MOR^wildtype^ and MOR^Y7.43A^ were expressed in the whole brain of mice (Supplementary Figure S2), indicating the mouse models were built successfully. MOR^wildtype^ partially rescued the fentanyl and morphine analgesia compared with the vector AAV group. The MOR^Y7.43A^ group significantly increased the %MPE of fentanyl compared with MOR^wildtype^, but there was no difference in the development of morphine analgesia (Figures 4E,F). Altogether, these observations revealed that the Y7.43 site was associated with fentanyl analgesia.

### Activation mechanism of *µ*-opioid receptor by fentanyl and morphine

The available results suggested that Y7.43 is a key residue for differential changes of downstream signal pathways of MOR stimulated by fentanyl and morphine. MOR^Y7.43A^ enhanced analgesia of fentanyl but not morphine. To investigate why Y7.43 had varying impacts on different types of MOR agonists, we used MD simulations to investigate the mechanism of fentanyl and morphine activating MOR-mediated signaling.

**FIGURE 5:**
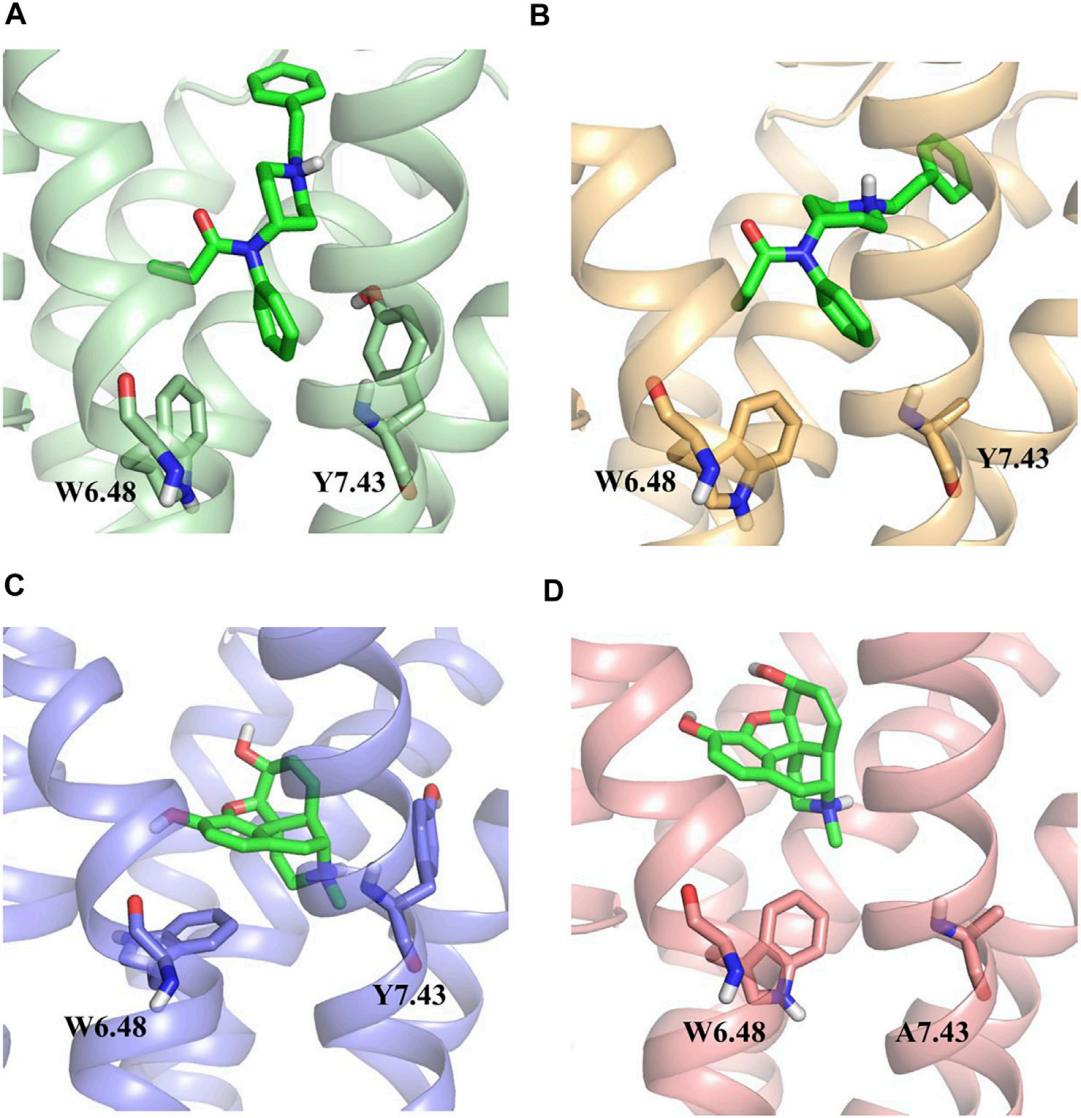
Hydrophobic interactions between W6.48 and Y7.43 during simulations. **(A,B)** hydrophobic interactions between W6.48 and Y7.43 during the binding process of fentanyl with µ-opioid receptor (MOR). Residues are shown in the ball–stick model; wild type (WT), green ribbon; Y7.43A, yellow ribbon. **(C,D)** hydrophobic interactions between W6.48 and Y7.43 during the binding process of morphine with MOR. Residues are shown in the ball–stick model; WT, light blue ribbon; Y7.43A, pink ribbon.

**FIGURE 6:**
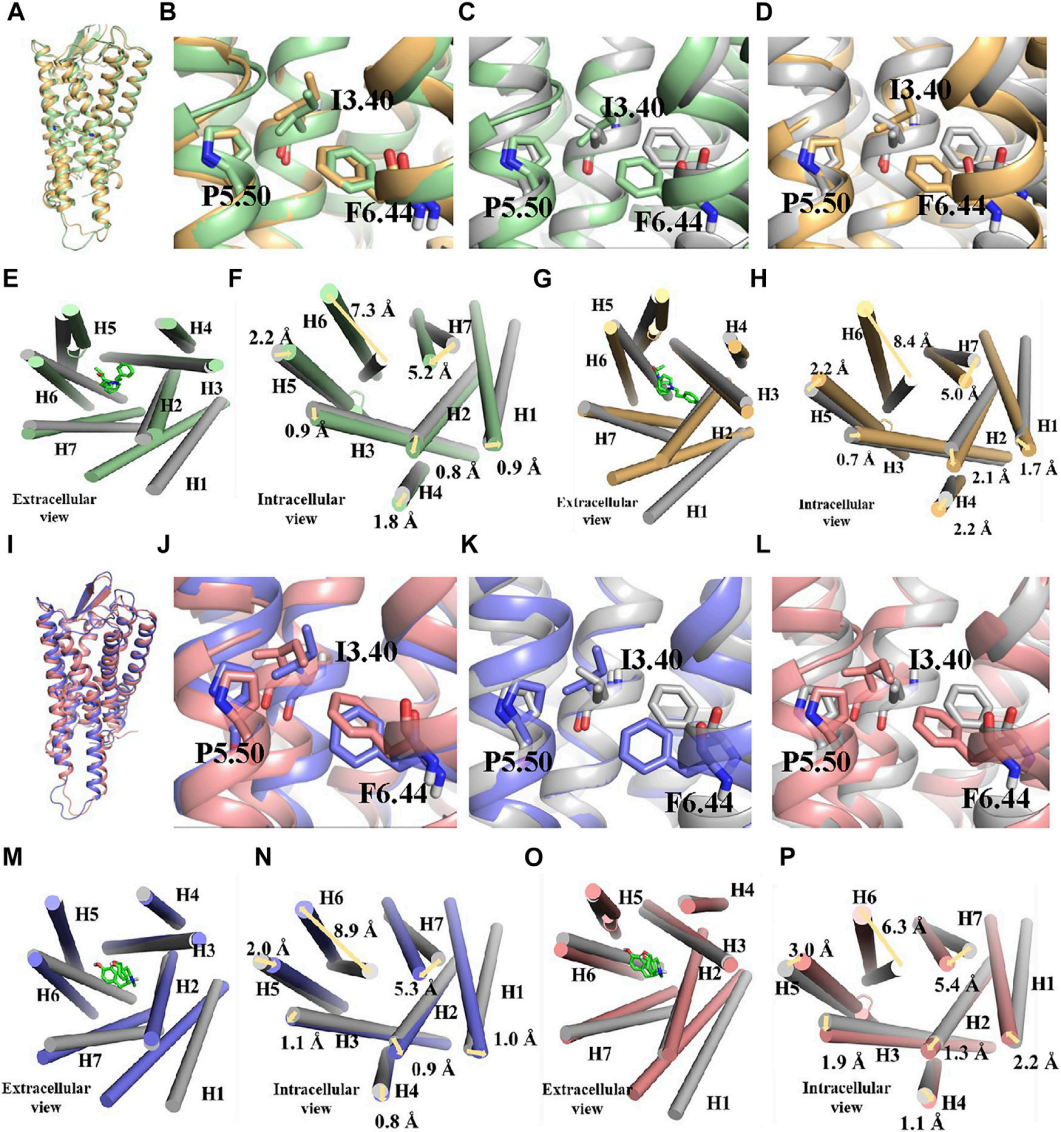
Conserved P^5.50^-I^3.40^-F^6.44^ triad rearrangement and transmembrane helix change during simulations. **(A–H)** binding poses and changes of transmembrane helix during the binding process of fentanyl with µ-opioid receptor (MOR). Active wild type (WT), green; active Y7.43A, yellow; apo state, gray; they are shown in ribbon and cylindrical shape in binding pose and transmembrane helix, respectively. Residues are shown in the ball–stick model. **(I–P)** binding poses and changes of transmembrane helix during the binding process of morphine with MOR. Active WT, blue; active Y7.43A, pink; apo state, gray; they are shown in ribbon and cylindrical shape in binding pose and transmembrane helix, respectively. Residues are shown in the ball–stick model.

The whole simulation process lasted for 100 ns, and all the systems remained stable (Supplementary Figure S2). In the fentanyl–receptor system, Y7.43 site mutation disrupted hydrophobic interactions between W6.48 and Y7.43 (Figures 5A,B). In terms of morphine–WT and morphine–Y7.43A systems, the hydrophobic interactions have been maintained (Figures 5C,D). In addition, the conserved P^5.50^-I^3.40^-F^6.44^ triad, an important activation switch that stayed at the bottom of the binding pocket of the agonist, rearranged after the binding of the agonist with MOR (Huang et al., 2015; Huang et al., 2021). As shown in Figure 6, F^6.44^ moved outwardly toward the cellular membrane (intracellular view), leading to the 7.3 and 8.4 Å outward movement of H6 in the fentanyl–WT and fentanyl–Y7.43A systems, respectively. This result suggested that the Y7.43 site mutation slightly increased the outward migration of H6 compared with WT. In the morphine–receptor activated system, H6 moved outward 6.3 Å after site mutation, which was less than 8.9 Å in the WT system (intracellular view). Besides, in the fentanyl–WT and fentanyl–Y7.43A systems, we also found the degree of I^3.40^ flipping was consistent compared to the apo state, while it varied in the two activated states of morphine binding with a receptor. In summary, the binding of fentanyl with MOR^Y7.43A^ broke up hydrophobic interactions and rearranged the conserved P^5.50^-I^3.40^-F^6.44^ triad. The rearrangement of the conserved P^5.50^-I^3.40^-F^6.44^ triad induced the outward movement of H6, which ultimately may lead to a differential impact on signal pathways by agonists stimulating the receptor.

## Discussion

In our studies, we first predicted the key amino acid residues playing an important role in agonists binding to the receptor by molecular docking. We then constructed MOR^Y7.43A^ to characterize the binding affinities and functional activities of fentanyl and morphine. The conventional hot plate test was used to determine the analgesic effect *in vivo* when the Y7.43 site was mutated. It showed that MOR^Y7.43A^ reduced the activation of G_i/o_ protein–dependent signaling, blocked *ß*-arrestin2 recruitment, and altered the kinetics of ERK1/2 phosphorylation induced by fentanyl. That is, MOR^Y7.43A^ delayed the occurrence of maximum ERK1/2 phosphorylation induced by fentanyl, compared with the MOR^wildtype^ group. It is worth noting that we observed that MOR^Y7.43A^ mice exhibited significantly greater analgesic potency of fentanyl than MOR^wildtype^. Moreover, compared with WT, Y7.43 site mutation broke up the hydrophobic interactions and promoted the outward movement of H6 in MOR stimulated by fentanyl but not morphine. Taken together, we show for the first time that Y7.43 site mutation reduced G_i/o_ protein signaling and blocked *ß*-arrestin2 recruitment, which may engage the enhanced analgesia induced by fentanyl.

Functional activity at MOR was characterized using cell-based assays designed to measure G_i/o_ protein signaling and *ß*-arrestin2 recruitment. In general, it is necessary to measure the affinity of MOR and ligands in determining the functional activity of MOR. In our research, we used HTRF competitive binding assay to determine the affinity of drugs in binding with MOR. Figure 2A shows the affinity of fluorescent ligand binding with MOR, and the K_d_ value was obtained from this curve. Figures 2B,C reflects the ability of drugs to compete with fluorescent ligand to bind to MOR, and the IC_50_ of drugs was obtained from this curve. The K_i_ value was calculated as the Cheng–Prusoff equation: $K_{i}=\frac{IC_{50}}{\left(1+\frac{\left[L\right]}{K_{d}}\right)}\;$ . The pK_i_ value directly reflects the affinity of drugs binding with MOR as shown in Table 1. From our results, there was no statistical difference between the relative affinity of morphine at MOR^Y7.43A^ and MOR^wildtype^. The relative affinity of fentanyl at MOR^Y7.43A^ was lower than that of MOR^wildtype^. The pK_i_ value of fentanyl was 91% of MOR^wildtype^ pK_i_. In combination with the practical application of Cisbio’s protocol, if the K_i_ value difference between changes is less than four times, it is usually considered that there is no difference in affinity. Therefore, we believe that the Y7.43 mutation almost did not change the affinity of the drugs in binding with MOR. On this basis, we monitored the activity of signaling pathways downstream of MOR.

Recent research has clarified how and where a ligand should bind with a receptor to cause adequate signaling activation. Y7.43 of MOR TM7 forms a ligand-binding pocket with W7.35 as highlighted by recent receptor crystallography data (Manglik et al., 2012; Huang et al., 2015) and previous mutagenesis and docking studies (Subramanian et al., 2000; Metzger et al., 2001). Molecular docking studies found that Y7.43 engaged in a hydrogen bond with D3.32. The interaction between D3.32 residue and the protonated nitrogen of fentanyl and morphine combined with hydrogen bond orients the molecules. These interactions have been proposed to be important for ligand-specific binding with MOR. In our results, receptor mutation functioned differently by fentanyl and morphine in signal transduction. MOR^Y7.43A^ did not change both cAMP and *ß*-arrestin2 signaling response by morphine, as it was similar to MOR^wildtype^. In complete contrast, decreased cAMP activity and no *ß*-arrestin2 activity were discovered at MOR^Y7.43A^ by fentanyl.

In addition, unremitting efforts are continuously put into research on the agonist-GPCR binding mode. In MD simulations, we also observed three other indexes except for hydrophobic interaction and P^5.50^-I^3.40^-F^6.44^ triad rearrangement, which were mentioned in the result section. The first index is a three to seven lock, which was formed by the hydrogen bond between D3.32 and Y7.43, and plays a key role in the activation of MOR (Cheng et al., 2018). As shown in Supplementary Figure S4, the hydrogen bonds were unstable and broken in fentanyl–WT and morphine–WT systems, respectively, and they disappeared because of the Y7.43 mutation in the fentanyl/morphine–Y7.43A system. Then, we monitored the ionic interaction between D3.32 and protonated nitrogen of agonists (Supplementary Figure S5), which facilitates the binding of an agonist with MOR (Pasternak and Pan, 2013; Huang et al., 2021). The third index is the hydrogen bond of T^6.34^-R^3.50^, which regulates conformation, and may stabilize the receptor in an inactive state (Jones et al., 1995; Lu et al., 1997; Rasmussen et al., 1999; Alewijnse et al., 2000; Scheer et al., 2000; Ohyama et al., 2002; Schneider et al., 2010; Manglik et al., 2012). As shown in Supplementary Figure S6, the hydrogen bonds were always broken during all stimulations in four systems. Therefore, the above three monitored indexes showed no difference in the binding process between agonists and receptors. Therefore, the hydrophobic interaction and the outward movement in H6 were significant clues in activating different signaling transduction mediated by fentanyl and morphine.

*β*-arrestin was previously thought to be the regulatory factor involved in endocytosis and desensitization of receptors (Luttrell and Lefkowitz, 2002). In studies spanning more than a decade, researchers have shown that the carboxyl-terminal phosphorylated MOR stimulated by agonists recruits *ß*-arrestin2, which may drive many of the pharmacological and unwanted side effects of MOR activation (Raehal et al., 2011; Bu et al., 2015). However, the molecular mechanisms initiating these effects remained unresolved. In our results, the *ß*-arrestin assay has revealed that Y7.43 site mutation was sufficient to abolish *ß*-arrestin2 recruitment. Fentanyl-enhanced analgesia was observed in MOR KO mice with rescued MOR^Y7.43A^, this could be because of the lack of *ß*-arrestin2 recruitment as the *ß*-arrestin assay showed. This is consistent with the conclusion that carboxyl-terminal phosphorylation site S375 mutation aimed at reducing *ß*-arrestin2 recruitment to MOR increased the analgesic potency of fentanyl but not morphine (Kliewer et al., 2019). Besides, *ß*-arrestin2 recruitment is also thought to be initiated by the phosphorylation of Tyrosine residues on the carboxyl-terminal tail following receptor activation. Next, we will focus on the relationship between Y7.43 and carboxy-terminal phosphorylation of MOR.

Morphine and fentanyl, as MOR agonists, were tested for analgesic potency at fixed doses. For the control JAX™C57BL/6J group, we found that fentanyl-induced analgesic rate peaked at 15 min after administration and disappeared after 135 min, compared with 30 and 150 min for morphine, respectively (Figures 4C,D). Fentanyl has a rapid onset and then rapid decay, whereas morphine has the opposite, which aligns with previous research in clinical profiles and animal studies (Gillis et al., 2020). It is of interest that MOR KO mice injected with AAV were sensitized when they were treated with fentanyl and morphine, which was completely different from MOR KO mice. Sensitivity and mania are expressed as jumping quickly in a hot plate apparatus and running circles in a cage. One plausible explanation for this difference is the stimulation of the AAV injection, and another possible explanation is the opioid-induced hyperalgesia (OIH) development in mice given repeated doses of fentanyl and morphine. Figures 4G,H show that basal pain thresholds were significantly decreased on days 3, 5, and 7 compared with that on day 1. Consistent with the existing literature, central sensitization is characterized by increased spontaneous activity and reduced thresholds (LaMotte et al., 1982; Coderre et al., 1993). Psychological factors, including anxiety and amplification of pain, could be modulating factors in the development of OIH (Lee and Yeomans, 2014).

Biased agonism has been proposed as a means to separate desirable from adverse drug responses downstream of MOR (Kenakin, 2011; Rankovic et al., 2016). The most accepted method for detection and quantification of biased agonism is the comparison of “transduction coefficients” based on the Black and Leff operational model of agonism (Black and Leff, 1983; Kenakin and Christopoulos, 2013). However, the contribution of the degree of bias to the improved separation of therapeutic benefit from side effects is unclear. A recent report has developed a novel idea that bias factor correlates with therapeutic window, which can be used to predict safer opioid analgesics (Schmid et al., 2017). In essence, the bias factor defines the extent of difference in relative agonist activity between two signaling assays (the bias factor is 10^ΔΔLogτ/KA(assay1−assay2) ) (Kenakin et al., 2012). The bias of a ligand is dependent upon the reference ligand and the type of assay used (De Neve et al., 2021). Bias factor calculations are no absolute values, but there are general trends. The higher the bias factor, the greater the separation between an agonist’s performance in the two assays relative to the performance of the reference agonist (Schmid et al., 2017). However, there was no reference agonist to evaluate the separation degree of two signaling pathways in our current results. It is impossible to calculate the bias factor. Our results showed that MOR^Y7.43A^ reduced the activity of the G_i/o_ protein pathway and blocked *ß*-arrestin2 recruitment stimulated by fentanyl. It enhanced fentanyl analgesia. Therefore, fentanyl analgesia may be mediated by both G_i/o_ protein and *ß*-arrestin signaling pathways. Besides, we also will focus on whether MOR^Y7.43A^ can reduce fentanyl-induced respiratory depression and constipation. Quantification of the contribution of signal pathway bias to therapeutic window separation remains to be studied. We look forward to a more comprehensive evaluation of how the signal pathway bias affects the therapeutic windows.

In summary, we indeed first elucidate the novel mechanisms of Y7.43 mutation affecting MOR signaling pathways. Further, functional selectivity in fentanyl and morphine can engage different mechanisms to induce analgesia. Through a better understanding of the molecular mechanisms of ligands specific binding with MOR, it is anticipated that we can bridge the knowledge gap on how chemical information encoded within the receptor regions’ controlled-effector coupling is decoded into the effect mediated by signaling pathways through the specific interactions it makes within the receptor binding site. Thus, by rationally designing novel compounds to interact predictably with MOR^Y7.43^, we might improve the chance of successfully designing new analgesics with fewer side effects and stronger analgesic ability.

## Data Availability

The datasets presented in this study can be found in online repositories. The names of the repository/repositories and accession number(s) can be found in the article/Supplementary Material.
