# Supplementary material for: Tyrosine 7.43 is important for mu-opioid receptor downstream signaling pathways activated by fentanyl
Source: Front Pharmacol. 2022 Sep 2;13:919325. doi: 10.3389/fphar.2022.919325 (PMC9478952; doi:10.3389/fphar.2022.919325)
Supplement: Supplementary file 1 [file DataSheet1.docx]

Supplementary Material


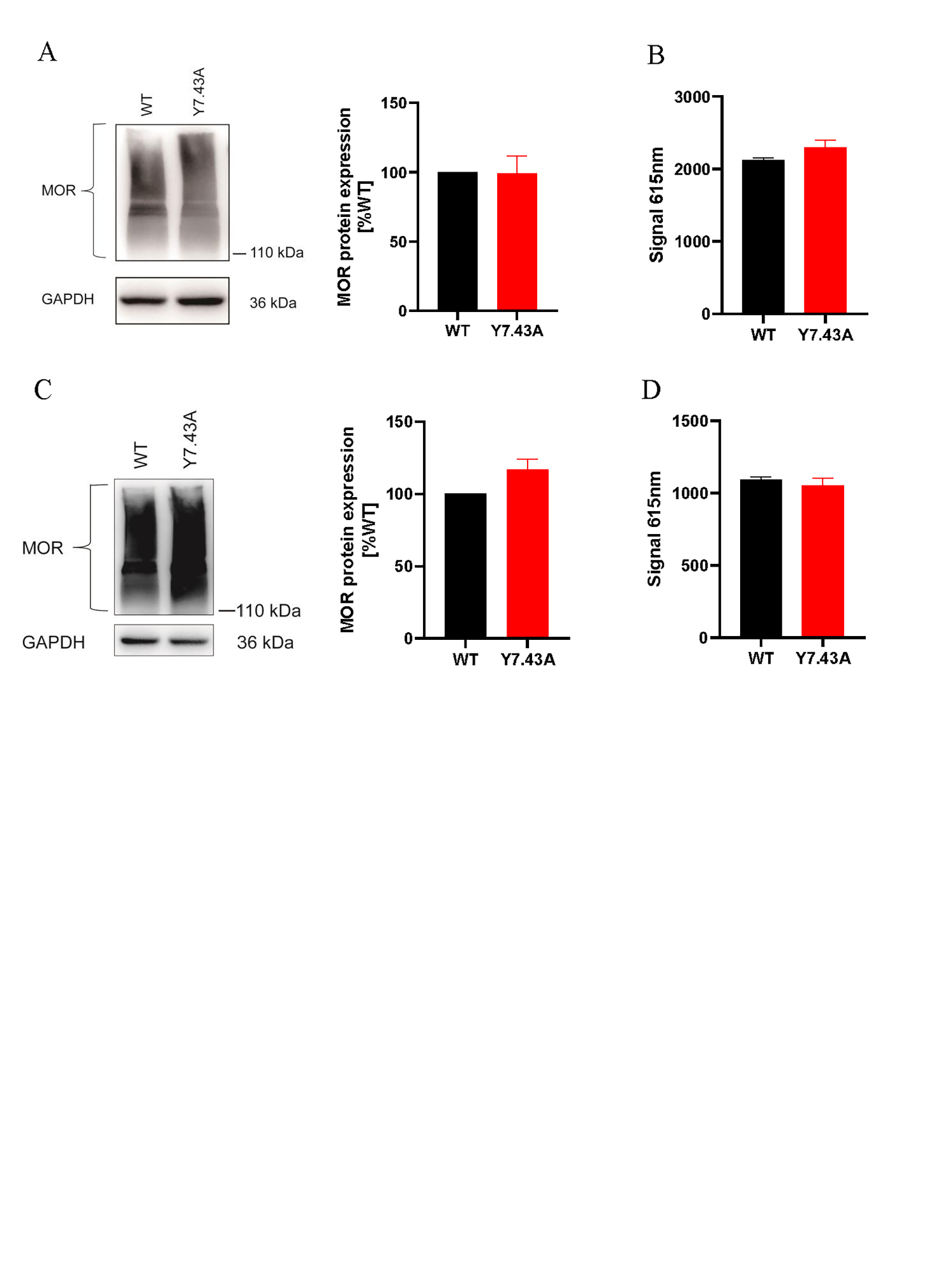
**Supplementary Figure 1.** WT and Y7.43A mutant MOR expression in HTRF competitive binding assay **(A,B)** and cAMP assay **(C,D)**. **(A,C)** Representative western blots and quantification of MOR. **(B,D)** Receptor cell surface expression by fluorescence labeling.

Data represented as mean ± SEM from three independent experiments; statistical significance of differences between WT and Y7.43A mutant MOR was analyzed with unpaired two-tailed *t*-test.


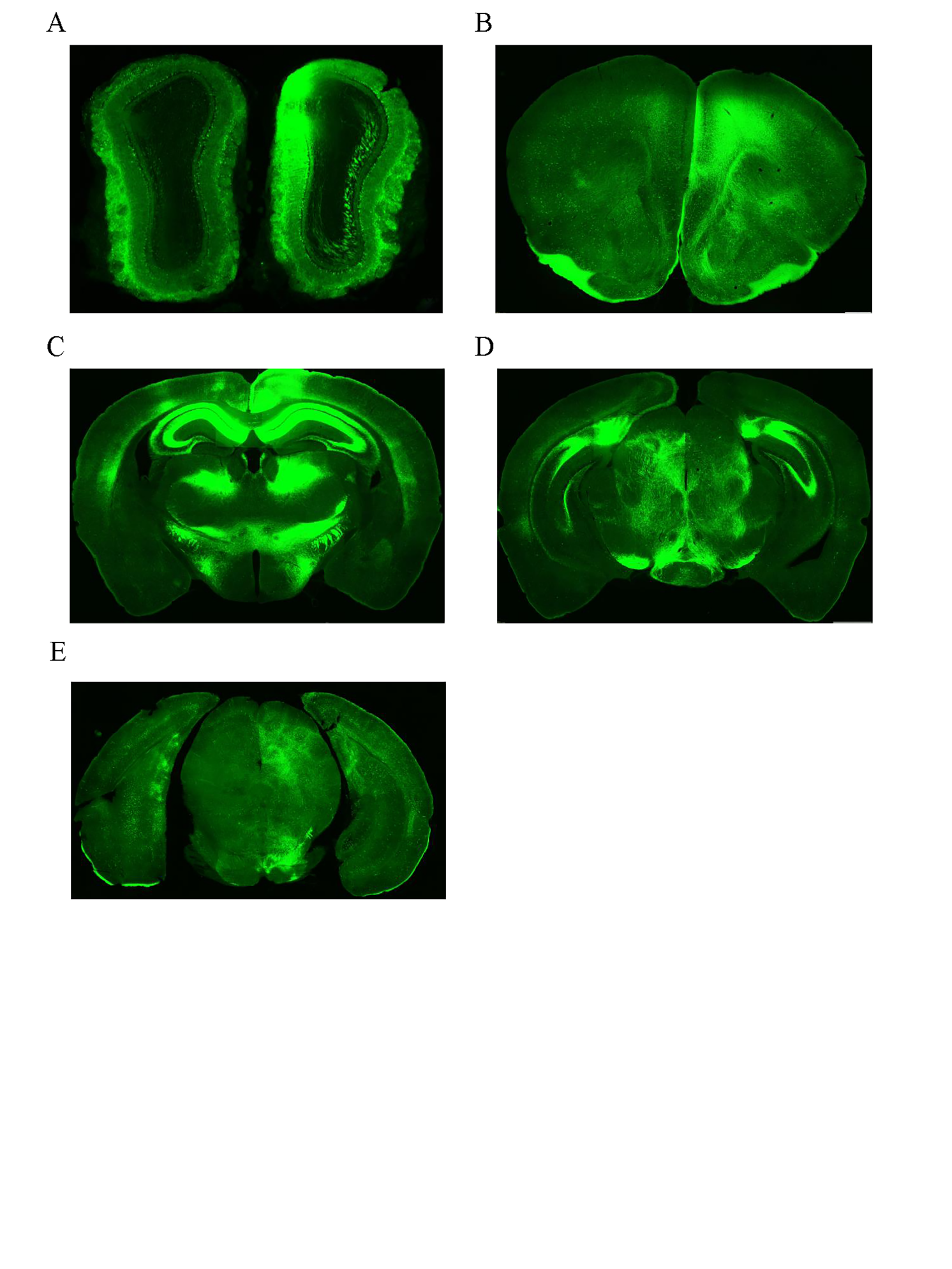


**Supplementary Figure 2.** The AAV expression of central nervous system (CNS) in MOR KO mice. MOR KO mice received a right cerebral lateral ventricle injection of vector/ MOR^wildtype^/MOR^Y7.43A^ virus. The dose was 2.5×10^10^ vg in each case. The virus expression was analyzed 21 days post-injection. Representative coronal brain sections were scanned using Olympus VS120. **(A)** Expression in olfactory bulb. **(B-E)** Cerebrum.


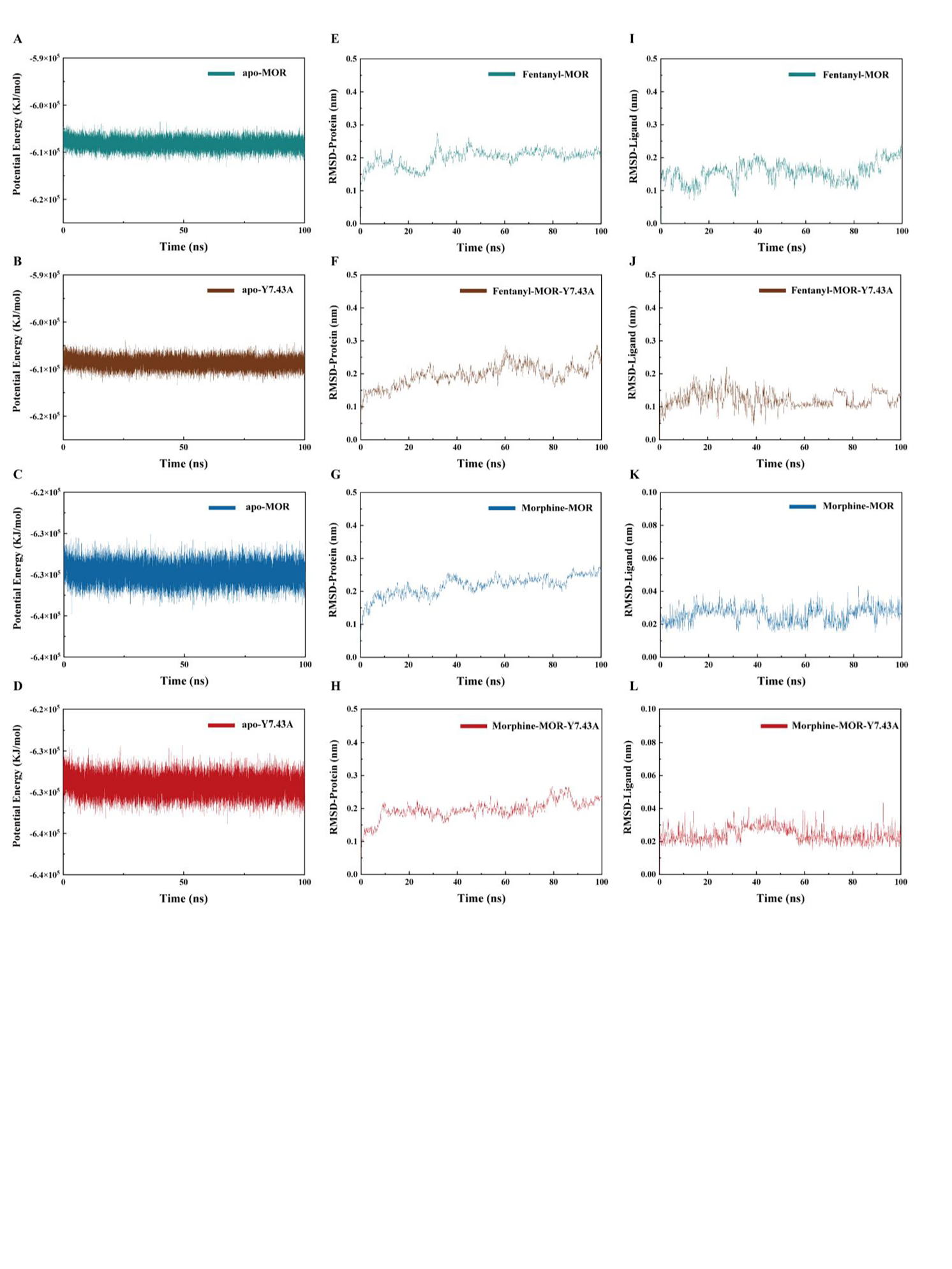


**Supplementary Figure 3.** The average potential energy and core C_α_ root mean-square deviation (RMSD) in molecular dynamics simulation system. **(A-D)** The average potential energy of apo states. **(E-H)** The receptor RMSD of active states in fentanyl/morphine-WT/Y7.43A system. **(I-L)** The ligand RMSD of active states in fentanyl/morphine-WT/Y7.43A system.


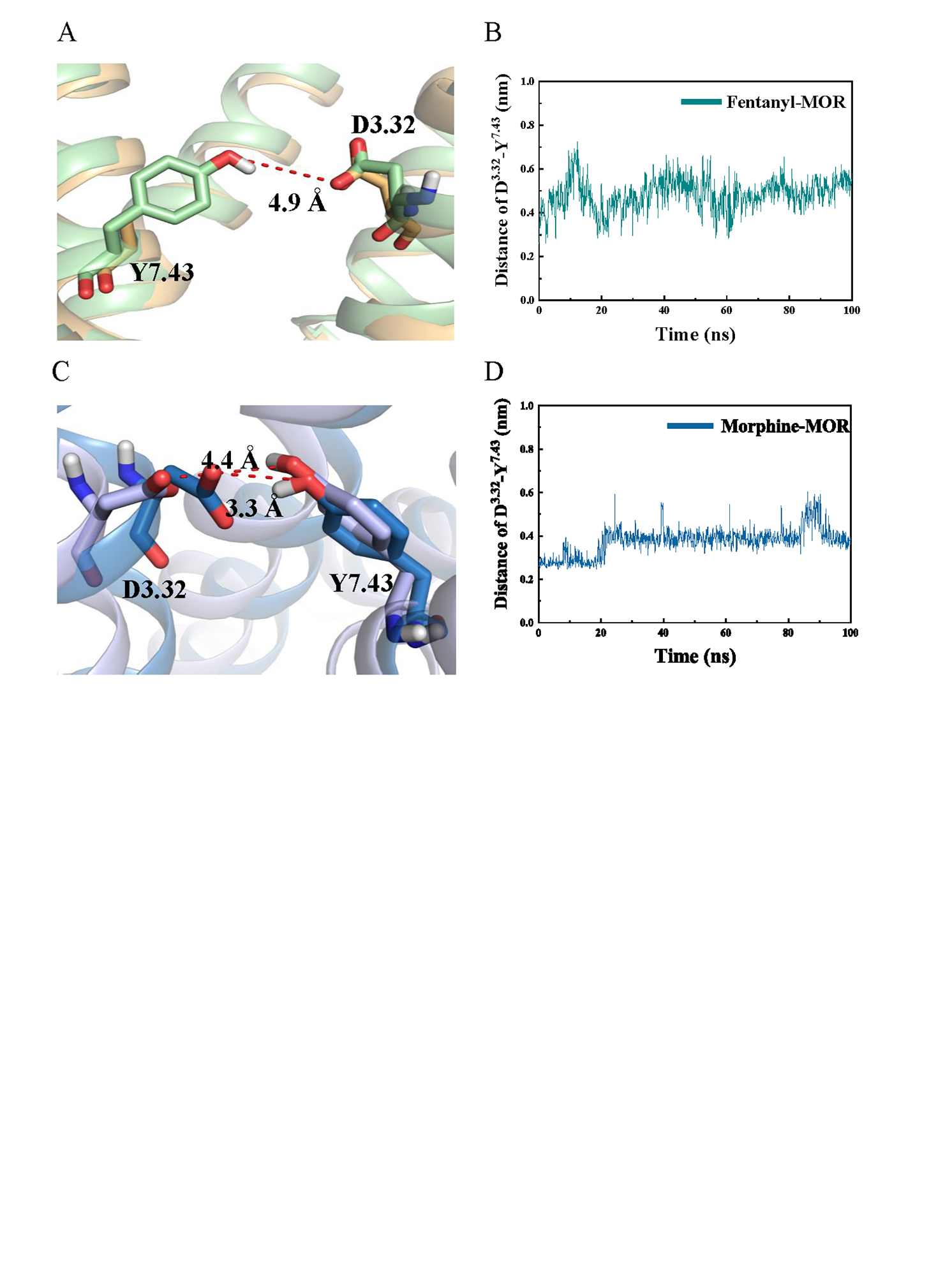


**Supplementary Figure 4.** Distance of D3.32 with Y7.43 during simulations. **(A,B)** Distance change of D3.32 with Y7.43 during the binding process of fentanyl with MOR. Residues are shown in the ball−stick model; WT, green ribbon; Y7.43A, yellow ribbon. **(C,D)** Distance change of D3.32 with Y7.43 during the binding process of morphine with MOR. **(C)** Comparison of interaction of D3.32 with Y7.43 before and after 21.4 ns. Residues are shown in the ball−stick model; 21.4 ns, blue ribbon; 21.5 ns, light blue ribbon.


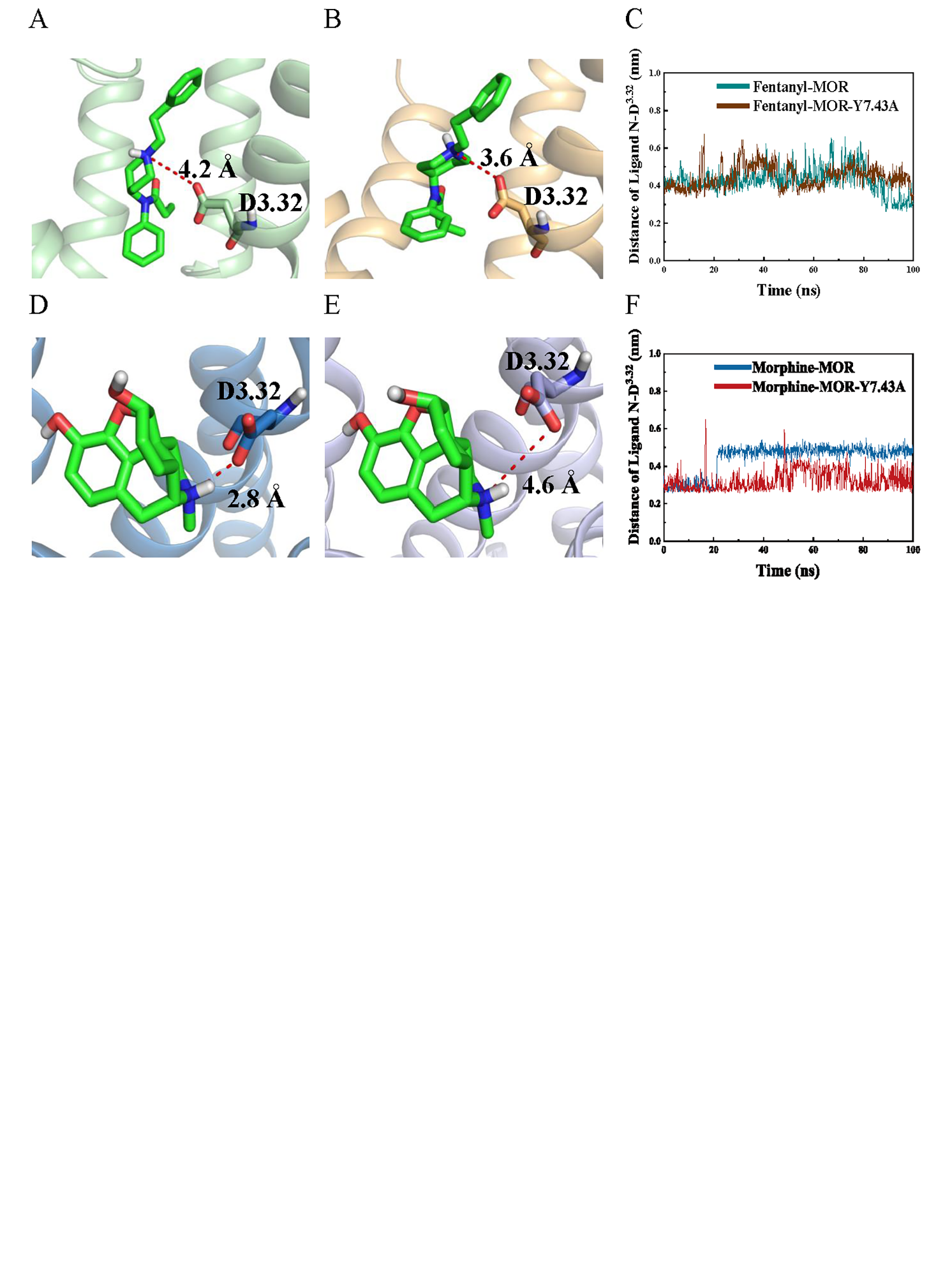


**Supplementary Figure 5.** Distance of ligand with D3.32 during simulations. **(A,B,C)** Distance change of protonated nitrogen of fentanyl with D3.32. Residues and fentanyl are shown in the ball−stick model, such as fentanyl is green. Light green and yellow ribbon are WT and Y7.43A, respectively. **(D,E,F)** Distance change of protonated nitrogen of morphine with D3.32. Morphine is shown in the green ball−stick model. **(D,E)** Comparison of interaction of morphine with D3.32 before and after 21.4 ns during the binding of morphine with WT. Residues are shown in the ball−stick model; 21.4 ns, blue ribbon; 21.5 ns, light blue ribbon.


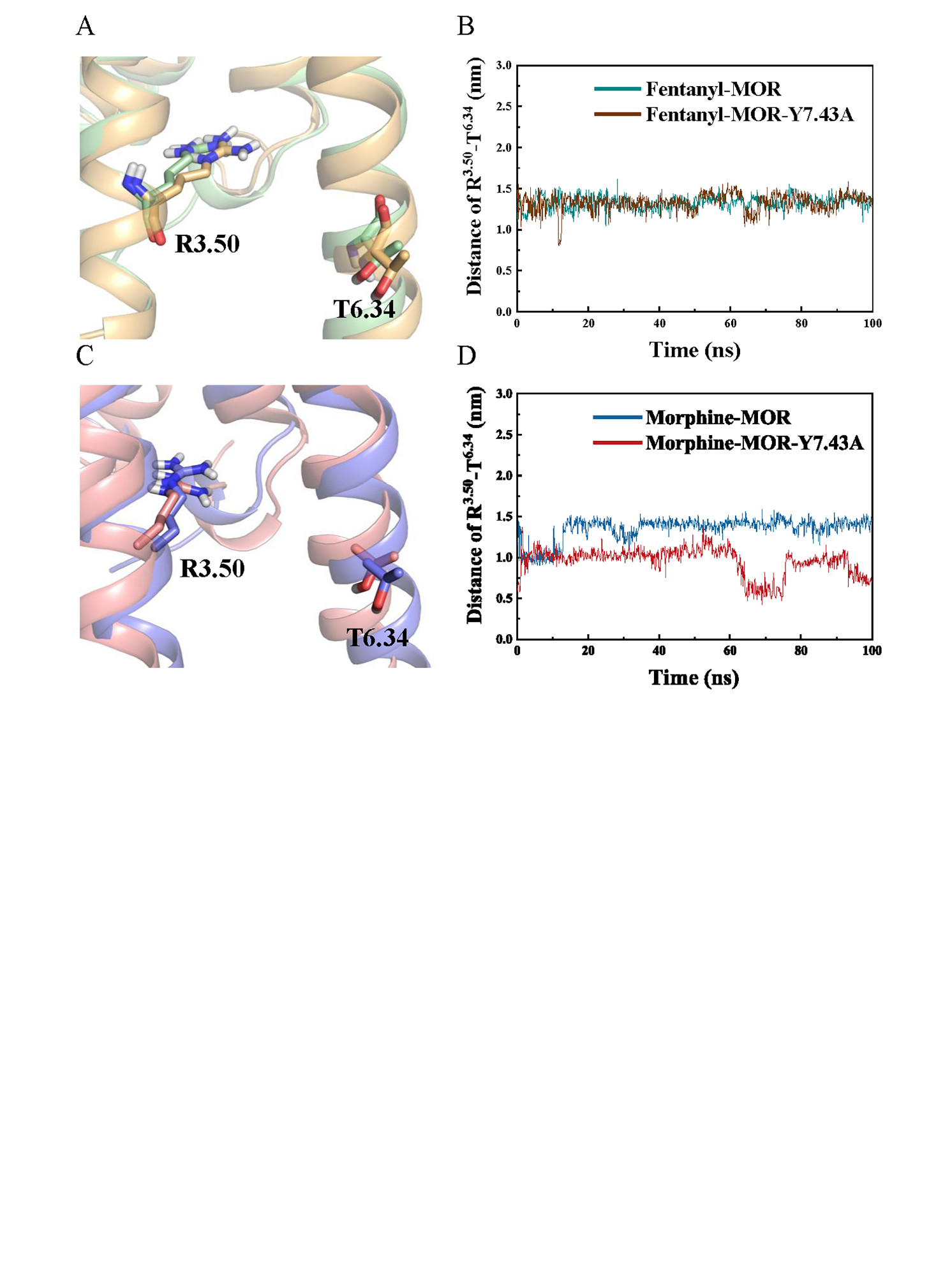


**Supplementary Figure 6.** Distance of R3.50 with T6.34 during simulations. **(A,B)** Distance change of R3.50 with T6.34 during the binding process of fentanyl with MOR. Residues are shown in the ball−stick model; WT, green ribbon; Y7.43A, yellow ribbon. **(C,D)** Distance change of R3.50 with T6.34 during the binding process of morphine with MOR. Residues are shown in the ball−stick model; WT, light blue ribbon; Y7.43A, pink ribbon.
